# SARS-CoV-2 Infection and Death Rates Among Maintenance Dialysis Patients During Delta and Early Omicron Waves — United States, June 30, 2021–September 27, 2022

**DOI:** 10.15585/mmwr.mm7232a4

**Published:** 2023-08-11

**Authors:** Jose Navarrete, Gregory Barone, Iram Qureshi, Austin Woods, Kira Barbre, Lu Meng, Shannon Novosad, Qunna Li, Minn Minn Soe, Jonathan Edwards, Emily Wong, Hannah E. Reses, Sydney Guthrie, John Keenan, Leticia Lamping, Meeyoung Park, Sorie Dumbuya, Andrea L. Benin, Jeneita Bell

**Affiliations:** ^1^Renal Division, Department of Medicine, Emory University School of Medicine, Atlanta, Georgia; ^2^Division of Healthcare Quality Promotion, National Center for Emerging and Zoonotic Infectious Diseases, CDC; ^3^Oak Ridge Institute for Science and Education, Oak Ridge, Tennessee; ^4^Leidos, Inc., Atlanta, Georgia; ^5^Chenega Enterprise Systems & Solutions, LLC, Chesapeake, Virginia; ^6^Goldbelt C6, Chesapeake, Virginia ^7^Division of Emergency Operations, Office of Readiness and Response, CDC.

**Summary**

**What is already known about this topic?**

Patients receiving maintenance dialysis are at increased risk for complications related to SARS-CoV-2 infection, including death.

**What is added by this report?**

During June 30, 2021–September 27, 2022, rates of SARS-CoV-2 infection and COVID-19–related death were higher among maintenance dialysis patients compared with rates in the U.S. population. These higher infection rates were attenuated by vaccination.

**What are the implications for public health practice?**

Patients receiving maintenance dialysis benefit from staying up to date with recommended COVID-19 vaccination. Continued efforts to mitigate transmission of respiratory viruses in dialysis facilities are warranted.

## Abstract

Persons receiving maintenance dialysis are at increased risk for SARS-CoV-2 infection and its severe outcomes, including death. However, rates of SARS-CoV-2 infection and COVID-19–related deaths in this population are not well described. Since November 2020, CDC’s National Healthcare Safety Network (NHSN) has collected weekly data monitoring incidence of SARS-CoV-2 infections (defined as a positive SARS-CoV-2 test result) and COVID-19–related deaths (defined as the death of a patient who had not fully recovered from a SARS-CoV-2 infection) among maintenance dialysis patients. This analysis used NHSN dialysis facility COVID-19 data reported during June 30, 2021–September 27, 2022, to describe rates of SARS-CoV-2 infection and COVID-19–related death among maintenance dialysis patients. The overall infection rate was 30.47 per 10,000 patient-weeks (39.64 among unvaccinated patients and 27.24 among patients who had completed a primary COVID-19 vaccination series). The overall death rate was 1.74 per 10,000 patient-weeks. Implementing recommended infection control measures in dialysis facilities and ensuring patients and staff members are up to date with recommended COVID-19 vaccination is critical to limiting COVID-19–associated morbidity and mortality.

## Introduction

Persons receiving maintenance dialysis are at increased risk for SARS-CoV-2 infection (*1*) and its severe outcomes, including death (*2*). However, rates of SARS-CoV-2 infection and COVID-19–related death among dialysis patients, and the impact of COVID-19 vaccination on these rates, are not well described. CDC’s National Healthcare Safety Network (NHSN) collects weekly facility-level data monitoring incidence of SARS-CoV-2 infection and death among maintenance dialysis patients.* During the COVID-19 Public Health Emergency, the Centers for Medicare & Medicaid Services instituted emergency requirements through the End-stage Renal Disease Network, mandating that COVID-19 cases, deaths, and vaccination status of dialysis facility patients and staff members be reported to NHSN.

## Methods

A SARS-CoV-2 infection was defined as any positive SARS-CoV-2 test result for a dialysis patient during the preceding 7 days. A COVID-19–related death was defined as a death occurring in a patient who had not fully recovered from a SARS-CoV-2 infection. Facility-level data on SARS-CoV-2 infections and deaths were stratified into waves (periods between weeks with the lowest infection rates among NHSN dialysis patients). Waves corresponded to the dominant circulating SARS-CoV-2 variant: Delta (June 30–October 26, 2021), first Omicron (October 27, 2021–March 22, 2022), and second Omicron (March 23–September 27, 2022). Pooled mean SARS-CoV-2 infection and death rates (events per 10,000 patient-weeks) among dialysis patients were calculated as the sum of weekly cases divided by the weekly patient census during each wave. COVID-19–related deaths were ascribed to the week during which the death occurred. The rates by wave, with 95% CIs, were calculated and stratified by rural-urban continuum code,^†^ county-level social vulnerability index tertiles (low, medium, and high),^§^ state, region,^¶^ dialysis facility size, and primary series and monovalent booster dose vaccination completion status. Age group–stratified COVID-19 rates among the U.S. population (cases per 10,000 population) were calculated as the total number of cases (by specific age group) reported during a week divided by the estimated age-specific U.S. population, using COVID-19 case surveillance public use data.** Analyses were performed using SAS software (version 9.4; SAS Institute). This activity was reviewed by CDC and was conducted consistent with applicable federal law and CDC policy.^††^

## Results

**FIGURE 1:**
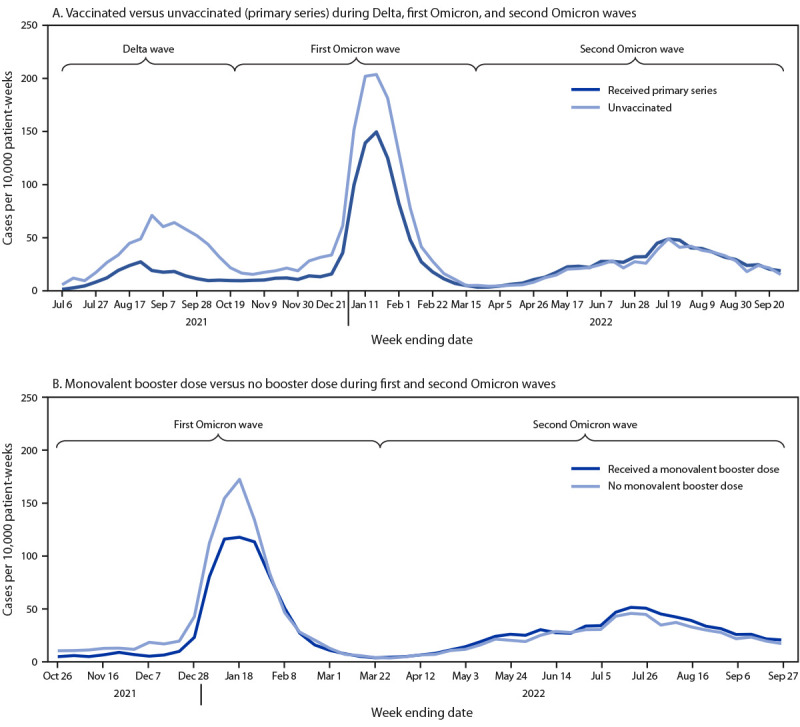
SARS-CoV-2 infections per 10,000 patient-weeks among maintenance dialysis patients, by COVID-19 primary (A) and booster dose (B) vaccination status — National Healthcare Safety Network, United States, June 30, 2021–September 27, 2022

A total of 7,848 dialysis facilities reported weekly SARS-CoV-2 infections and COVID-19–related deaths among 518,798 patients to NHSN during June 30, 2021–September 27, 2022. The overall pooled mean SARS-CoV-2 infection rate among maintenance dialysis patients was 30.47 per 10,000 patient-weeks, with a pooled mean COVID-19–related death rate of 1.74 per 10,000 patient-weeks (Table). The highest infection and death rates were observed during the first Omicron wave (Figure 1).

**TABLE:** Pooled mean SARS-CoV-2 incidence and COVID-19–associated death rates* per 10,000 patient-weeks among maintenance dialysis patients during each COVID-19 wave,^†^ by region, urbanicity, social vulnerability index, facility size, primary vaccination status, and monovalent booster dose receipt status — National Healthcare Safety Network, United States, June 30, 2021–September 27, 2022

Characteristic	SARS-CoV-2 incidence, by wave (95% CI)				COVID-19–associated death rates, by wave (95% CI)			
	Overall	Delta	First Omicron	Second Omicron	Overall	Delta	First Omicron	Second Omicron
**Overall^§^**	**30.47 (29.02–31.97)**	**20.13 (18.99–21.36)**	**46.45 (44.64–48.30)**	**25.05 (23.74–26.44)**	**1.74 (1.44–2.12)**	**1.96 (1.62–2.34)**	**2.66 (2.26–3.11)**	**0.59 (0.43–0.81)**
**Region^¶^**								
Midwest	**27.64 (27.26–28.02)**	16.92 (16.29–17.56)	52.48 (51.47–53.51)	23.55 (22.95–24.16)	**1.65 (1.56–1.75)**	1.43 (1.25–1.62)	3.52 (3.26–3.79)	0.54 (0.45–0.64)
Mountain	**28.12 (27.45–28.81)**	24.35 (23.03–25.72)	51.81 (50.04–53.62)	22.02 (21.00–23.08)	**1.89 (1.72–2.07)**	1.91 (1.56–2.31)	4.12 (3.64–4.65)	0.66 (0.50–0.86)
Northeast	**28.26 (27.83–28.70)**	9.90 (9.37–10.46)	52.72 (51.57–53.89)	28.87 (28.11–29.64)	**1.63 (1.53–1.74)**	1.00 (0.83–1.18)	2.90 (2.64–3.18)	0.87 (0.75–1.01)
Pacific	**24.71 (24.34–25.09)**	13.31 (12.74–13.91)	41.54 (40.61–42.49)	29.28 (28.57–30.00)	**1.01 (0.94–1.09)**	1.19 (1.02–1.37)	1.83 (1.65–2.04)	0.44 (0.36–0.53)
South	**26.11 (25.87–26.35)**	26.60 (26.09–27.12)	43.39 (42.79–43.99)	21.63 (21.25–22.01)	**1.68 (1.62–1.74)**	2.74 (2.58–2.91)	2.48 (2.34–2.63)	0.54 (0.48–0.60)
Noncontiguous	**43.56 (41.55–45.64)**	40.00 (36.01–44.31)	52.40 (48.18–56.89)	58.45 (54.48–62.63)	**1.57 (1.22–2.00)**	3.36 (2.31–4.74)	1.60 (0.96–2.51)	0.96 (0.54–1.60)
Urbanicity**^,††^								
Large core metro	**28.33 (27.26–28.02)**	16.16 (16.29–17.56)	45.03 (51.47–53.51)	23.02 (22.95–24.16)	**1.26 (1.56–1.75)**	1.37 (1.25–1.62)	2.19 (3.26–3.79)	0.45 (0.45–0.64)
Large fringe metro	**28.14 (27.45–28.81)**	16.33 (23.03–25.72)	43.78 (50.04–53.62)	23.53 (21.00–23.08)	**1.41 (1.72–2.07)**	1.49 (1.56–2.31)	2.49 (3.64–4.65)	0.51 (0.50–0.86)
Medium metro	**33.16 (27.83–28.70)**	24.49 (9.37–10.46)	48.40 (51.57–53.89)	26.75 (28.11–29.64)	**1.84 (1.53–1.74)**	2.36 (0.83–1.18)	2.88 (2.64–3.18)	0.67 (0.75–1.01)
Small metro	**32.78 (24.34–25.09)**	25.43 (12.74–13.91)	48.64 (40.61–42.49)	25.14 (28.57–30.00)	**2.15 (0.94–1.09)**	2.92 (1.02–1.37)	3.40 (1.65–2.04)	0.66 (0.36–0.53)
Rural	**35.70 (25.87–26.35)**	27.66 (26.09–27.12)	52.62 (42.79–43.99)	27.73 (21.25–22.01)	**2.62 (1.62–1.74)**	3.75 (2.58–2.91)	3.94 (2.34–2.63)	0.85 (0.48–0.60)
Noncore	**34.59 (41.55–45.64)**	27.09 (36.01–44.31)	49.66 (48.18–56.89)	27.69 (54.48–62.63)	**2.39 (1.22–2.00)**	3.43 (2.31–4.74)	3.48 (0.96–2.51)	0.83 (0.54–1.60)
**SVI^§§^**								
Low	**30.92 (30.57–31.27)**	18.21 (17.69–18.74)	46.93 (46.17–47.69)	26.55 (26.04–27.06)	**1.64 (1.56–1.72)**	1.75 (1.59–1.92)	2.83 (2.65–3.03)	0.61 (0.54–0.70)
Medium	**30.99 (30.66–31.32)**	21.02 (20.51–21.55)	47.37 (46.66–48.10)	24.58 (24.12–25.05)	**1.77 (1.69–1.85)**	2.06 (1.90–2.23)	2.93 (2.76–3.12)	0.65 (0.58–0.72)
High	**30.06 (29.76–30.37)**	21.23 (20.73–21.73)	45.74 (45.07–46.41)	23.43 (23.01–23.86)	**1.59 (1.53–1.67)**	2.25 (2.09–2.41)	2.44 (2.29–2.59)	0.50 (0.44–0.56)
**Facility size^¶¶^**								
Small	**32.50 (32.13–32.88)**	23.28 (22.68–23.89)	48.88 (48.08–49.69)	25.63 (25.12–26.15)	**1.66 (1.58–1.75)**	1.91 (1.74–2.09)	2.81 (2.62–3.01)	0.60 (0.52–0.68)
Medium	**30.30 (29.95–30.67)**	20.53 (19.97–21.11)	46.21 (45.43–46.99)	24.16 (23.66–24.66)	**1.66 (1.58–1.75)**	2.02 (1.84–2.20)	2.78 (2.59–2.97)	0.55 (0.48–0.63)
Large	**30.28 (29.99–30.57)**	18.38 (17.95–18.82)	46.09 (45.46–46.72)	25.57 (25.15–25.99)	**1.65 (1.58–1.72)**	1.93 (1.79–2.07)	2.68 (2.53–2.83)	0.63 (0.57–0.70)
Primary vaccination status***								
Full primary series	**27.24 (25.65–28.90)**	13.10 (12.00–14.28)	40.89 (38.91–42.91)	25.10 (23.58–26.71)	**—**	—	—	—
Not vaccinated	**39.64 (36.60–42.91)**	36.12 (33.39–39.05)	61.86 (57.90–66.08)	23.91 (21.50–26.60)	**—**	—	—	—
**Monovalent booster dose status^†††^**								
Full primary series and ≥1 booster dose	**30.62 (28.24–33.21)**	—	38.32 (35.16–41.62)	26.70 (24.62–28.86)	**—**	—	—	—
No booster dose	**33.69 (31.27–36.24)**	—	42.21 (39.76–44.80)	22.93 (20.75–25.30)	**—**	—	—	—

**Abbreviations**: NHSN = National Healthcare Safety Network; SVI = social vulnerability index.

* Cases and deaths per 10,000 patient-weeks. COVID-19–related deaths were defined as those among patients who died before fully recovering from SARS-CoV-2 infection; NHSN receives aggregate facility-level data; therefore, death rates could not be calculated by vaccination status.

^†^ Delta (June 30–October 26, 2021), first Omicron (October 27, 2021–March 22, 2022), and second Omicron (March 23–September 27, 2022).

^§^ Pooled rates within each category might differ from overall rates because of smaller sized subcategories.

^¶^ *South:* Alabama, Arkansas, Delaware, Florida, Georgia, Kentucky, Louisiana, Maryland, Mississippi, North Carolina, Oklahoma, South Carolina, Tennessee, Texas, Virginia, West Virginia, and District of Columbia; *Midwest:* Ilinois, Indiana, Iowa, Kansas, Michigan, Minesota, Missouri, Nebraska, North Dakota, Ohio, South Dakota, and Wisconsin; *Mountain:* Arizona, Colorado, Idaho, Montana, New Mexico, Nevada, Utah, and Wyoming; *Pacific:* California, Oregon, and Washington; *Northeast:* Connecticut, Maine, Massachusetts, New Hampshire, New Jersey, New York, Pennsylvania, Rhode Island, and Vermont; *Noncontiguous:* Alaska and Hawaii.

** Washington, DC, Puerto Rico, and U.S. Virgin Islands were not included in urbanicity subanalysis.

^††^ https://www.ers.usda.gov/data-products/rural-urban-continuum-codes

^§§^ https://www.atsdr.cdc.gov/placeandhealth/svi/index.html

^¶¶^ Small, medium, and large facilities are defined as having 0–16, 17–22, and ≥23 dialysis stations, respectively.

*** Primary vaccination data were available during all three waves (June 30, 2021–September 27, 2022).

^†††^ Monovalent booster dose vaccination data were only available during first and second Omicron waves (October 27, 2021–September 27, 2022).

The overall incidence of SARS-CoV-2 infection among unvaccinated dialysis patients was 39.64 per 10,000 patient-weeks, compared with 27.24 per 10,000 among those who had received a complete primary COVID-19 vaccination series (Table). During the first and second Omicron waves (October 27, 2021–March 22, 2022), the overall infection rate among dialysis patients who had received ≥1 monovalent booster dose was 30.62, compared with 33.69 among vaccinated patients who had not received a monovalent booster dose. During the Delta and first Omicron waves, the infection rate among vaccinated patients was lower than that among unvaccinated patients (Figure 1), and during the first Omicron wave, the infection rate was lower among patients who had received a monovalent booster dose than among those who had not.

**FIGURE 2:**
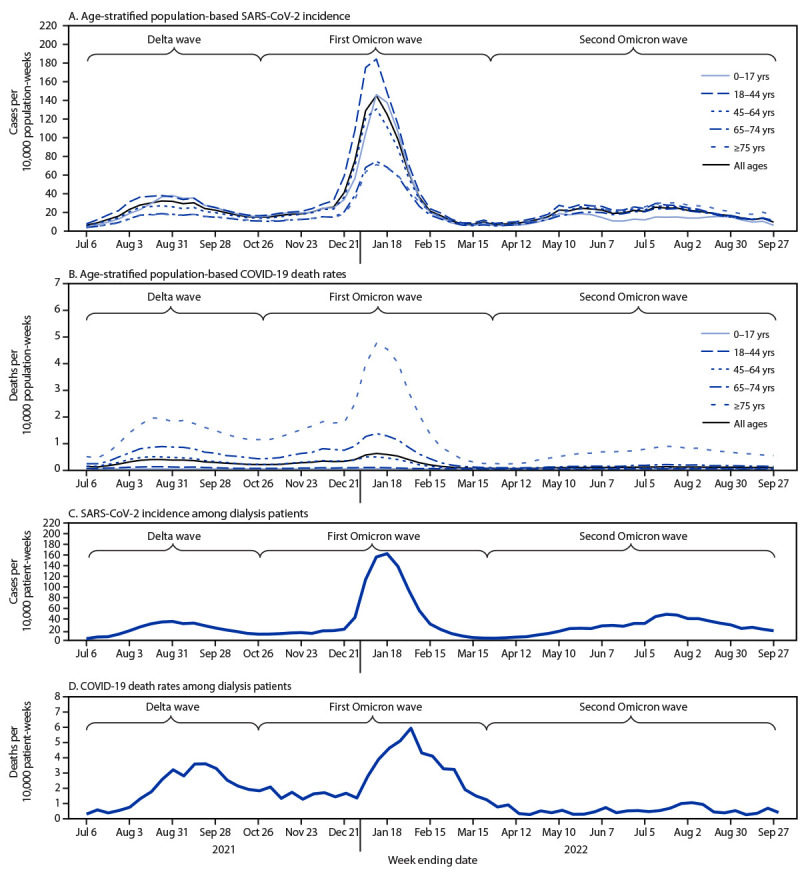
Age-stratified population-based SARS-CoV-2 incidence (A) and COVID-19–related death rates (B) among the overall U.S. population* and SARS-CoV-2 incidence (C) and COVID-19–related deaths (D)^†^ among maintenance dialysis patients^§^ — United States, June 30, 2021–September 27, 2022 * COVID-19 case surveillance public use data. https://data.cdc.gov/Case-Surveillance/COVID-19-Case-Surveillance-Public-Use-Data/vbim-akqf ^†^ COVID-19–related deaths were defined as those among patients who died before fully recovering from SARS-CoV-2 infection. ^§^ Data source: National Healthcare Safety Network.

Among the U.S. population, SARS-CoV-2 infection and death rates varied by age group, and the differences were most pronounced during the first Omicron wave (Figure 2). The SARS-CoV-2 infection rate in the U.S. population was 20.73 per 10,000 population-weeks during the Delta wave, 43.62 per 10,000 population-weeks during the first Omicron wave, and 17.13 per 10,000 population-weeks during the second Omicron wave. COVID-19–related death rates in the U.S. population were 0.24 per 10,000 population-weeks during the Delta wave, 0.26 per 10,000 population-weeks during the first Omicron wave, and 0.06 per 10,000 population-weeks during the second Omicron wave. The infection and death rates among maintenance dialysis patients followed similar patterns over time to those in the overall U.S. population (Figure 2).

## Discussion

During June 30, 2021–September 27, 2022, the overall SARS-CoV-2 infection rate among maintenance dialysis patients was 30.47 per 10,000 patient-weeks. During the Delta and first Omicron waves, differences in SARS-CoV-2 infection rates between vaccinated and unvaccinated dialysis patients were identified, a finding that has not been well documented in the literature for this population (*3*). However, no difference in infection rate among those who were vaccinated and unvaccinated was noted during the second Omicron wave. This might be because of lower overall infection rates and declining vaccine effectiveness over time, as well as the emergence of new variants (*4*). Although formal studies of vaccine effectiveness have not been conducted in this population, data suggest that receipt of a 2-dose primary mRNA COVID-19 vaccination series is protective in dialysis patients despite their having a slightly attenuated immune response (*5*). Approximately 70% of dialysis patients have completed a primary vaccination series, but only 54% received additional primary or booster doses, indicating substantial potential for improvement in vaccination coverage.^§§^ The reported side effects of SARS-CoV-2 vaccination did not differ between dialysis patients and persons not receiving dialysis (*6*). The need for patient education, efforts to combat vaccine misinformation, and on-site vaccination at dialysis facilities is ongoing.

The SARS-CoV-2 infection rate among both dialysis patients and the overall U.S. population was highest during the first Omicron wave. However, the infection rate among dialysis patients was mitigated by primary series vaccination, despite concerns about an attenuated immune response to vaccines among patients receiving dialysis. Although the SARS-CoV-2 infection rates were similar among dialysis patients and the U.S. population, patients receiving dialysis are generally older (*1*), and the infection rate among dialysis patients was higher than that among the U.S. population aged >65 years. The COVID-19–related death rate among dialysis patients was higher than that among the U.S. population with the highest death rates (i.e., persons aged >75 years). Compared with the U.S. population, patients receiving dialysis likely had higher rates of both SARS-CoV-2 infection and COVID-19–related death.

Most patients receiving dialysis must visit dialysis facilities to receive lifesaving treatment, which is performed in close proximity to other patients and facility staff members, three times each week. Many patients rely on shared transportation (e.g., public transit or medical transport van), and approximately 7% live in long-term care facilities (*6*), placing these persons at particularly high risk for infection and death related to COVID-19 (*7*). The infection rate among persons receiving dialysis can be reduced by adherence to recommended infection prevention practices, including early detection of symptomatic illness, appropriate location of infected patients during in-facility dialysis treatments, correct use of personal protective equipment, and implementation of protocols to safely discontinue transmission-based precautions for affected patients (*8*,*9*). Using engineering controls, including barriers between patients and improved ventilation and indoor air quality, might further reduce exposure to COVID-19 and other respiratory viruses.^¶¶^

### Limitations

The findings in this report are subject to at least five limitations. First, this report included data submitted by outpatient facilities to NHSN. Although the dataset included over 90% of the estimated total maintenance dialysis patients in the United States (*1*), patients receiving inpatient dialysis, home hemodialysis, and peritoneal dialysis might be underrepresented in this analysis. Second, facilities self-report data to NHSN, which might limit the validity of the information submitted. Third, the NHSN definition of a COVID-19–related death was not limited to a death in which COVID-19 was listed as a cause of death on the death certificate or one that occurred during a specific time frame after COVID-19 infection. Therefore, it is possible that some deaths were misclassified as COVID-19–related deaths, resulting in an inflated COVID-19–related death rate. Fourth, NHSN received aggregate facility-level data. Therefore, death rates could not be calculated by vaccination status, nor could patient-level covariates, including time since vaccination, previous COVID-19 infection, age, ethnicity, or comorbidities that play a role in the high death rate of patients receiving dialysis be considered. Finally, this analysis did not account for differences in COVID-19 testing and reporting between dialysis patients and the U.S. population. It is possible that a higher rate of COVID-19 testing among dialysis patients (*9*) might have affected the results.

### Implications for Public Health Practice

These findings underscore the need for dialysis patients and staff members to stay up to date with primary COVID-19 vaccine and booster dose recommendations*** and for dialysis facilities to implement effective infection control strategies^†††^ (*10*). To protect patients from SARS-CoV-2 and other respiratory viruses, facilities should continue to adhere to recommended infection prevention practices and work to improve facility design and layout.

## Footnotes

*
https://www.cdc.gov/nhsn/pdfs/newsletters/dec20-nl-508.pdf

†
https://www.ers.usda.gov/data-products/rural-urban-continuum-codes

§
https://www.atsdr.cdc.gov/placeandhealth/svi/index.html

¶ *South:* Alabama, Arkansas, Delaware, Florida, Georgia, Kentucky, Louisiana, Maryland, Mississippi, North Carolina, Oklahoma, South Carolina, Tennessee, Texas, Virginia, West Virginia, and District of Columbia; *Midwest*: Ilinois, Indiana, Iowa, Kansas, Michigan, Minesota, Missouri, Nebraska, North Dakota, Ohio, South Dakota, and Wisconsin; *Mountain*: Arizona, Colorado, Idaho, Montana, New Mexico, Nevada, Utah, and Wyoming; *Pacific:* California, Oregon, and Washington; *Northeast:* Connecticut, Maine, Massachusetts, New Hampshire, New Jersey, New York, Pennsylvania, Rhode Island, and Vermont; *Noncontiguous:* Alaska and Hawaii.

**
https://data.cdc.gov/Case-Surveillance/COVID-19-Case-Surveillance-Public-Use-Data/vbim-akqf

†† 45 C.F.R. part 46, 21 C.F.R. part 56; 42 U.S.C. Sect. 241(d); 5 U.S.C. Sect. 552a; 44 U.S.C. Sect. 3501 et seq.

§§
https://www.cdc.gov/nhsn/covid19/dial-vaccination-dashboard.html

¶¶
https://www.ashrae.org/technical-resources/bookstore/health-care-facilities-resources

***
https://www.cdc.gov/coronavirus/2019-ncov/vaccines/stay-up-to-date.html

†††
https://www.cdc.gov/coronavirus/2019-ncov/hcp/infection-control-recommendations.html?CDC_AA_refVal
